# Carnosine increases insulin-stimulated glucose uptake and reduces methylglyoxal-modified proteins in type-2 diabetic human skeletal muscle cells

**DOI:** 10.1007/s00726-022-03230-9

**Published:** 2023-01-13

**Authors:** Joseph J. Matthews, Mark D. Turner, Livia Santos, Kirsty J. Elliott-Sale, Craig Sale

**Affiliations:** 1 Sport, Health and Performance Research Centre, Musculoskeletal Physiology Research Group, School of Science and Technology, Nottingham Trent University, Nottingham, UK; 2 Department of Sport and Exercise, Centre for Life and Sport Sciences (CLaSS), Birmingham City University, Birmingham, UK; 3 Centre for Diabetes, Chronic Diseases & Ageing, School of Science and Technology, Nottingham Trent University, Nottingham, UK; 4 Institute of Sport, Manchester Metropolitan University, Manchester, UK

**Keywords:** Diabetes, Prediabetes, Glycaemia, Metabolism, Therapeutics

## Abstract

Type-2 diabetes (T2D) is characterised by a dysregulation of metabolism, including skeletal muscle insulin resistance, mitochondrial dysfunction, and oxidative stress. Reactive species, such as methylglyoxal (MGO) and 4-hydroxynonenal (4-HNE), positively associate with T2D disease severity and can directly interfere with insulin signalling and glucose uptake in skeletal muscle by modifying cellular proteins. The multifunctional dipeptide carnosine, and its rate-limiting precursor *β*-alanine, have recently been shown to improve glycaemic control in humans and rodents with diabetes. However, the precise mechanisms are unclear and research in human skeletal muscle is limited. Herein, we present novel findings in primary human T2D and lean healthy control (LHC) skeletal muscle cells. Cells were differentiated to myotubes, and treated with 10 mM carnosine, 10 mM *β*-alanine, or control for 4-days. T2D cells had reduced ATP-linked and maximal respiration compared with LHC cells (*p* = 0.016 and *p* = 0.005). Treatment with 10 mM carnosine significantly increased insulin-stimulated glucose uptake in T2D cells (*p* = 0.047); with no effect in LHC cells. Insulin-stimulation increased MGO-modified proteins in T2D cells by 47%; treatment with carnosine attenuated this increase to 9.7% (*p* = 0.011). There was no effect treatment on cell viability or expression of other proteins. These findings suggest that the beneficial effects of carnosine on glycaemic control may be explained by its scavenging actions in human skeletal muscle.

**Supplementary Information:**

The online version contains supplementary material available at 10.1007/s00726-022-03230-9.

## Introduction

Diabetes is a major public health problem; worldwide estimates show that 537 million people aged 20–79 years were living with diabetes in 2021, equivalent to 10.5% of the global adult population (Sun et al. 2022), with type-2 diabetes (T2D) accounting for > 90% of cases. T2D is characterised by a dysregulation of metabolism due to impaired insulin secretion, insulin resistance, or a combination of both (DeFronzo et al. 2015), which develops from impaired fasting glucose or impaired glucose tolerance (also known as prediabetes). Skeletal muscle insulin resistance and mitochondrial dysfunction are hallmarks of T2D, and although the aetiology is multifactorial, emerging research implicates oxidative and carbonyl stress as causative factors (Mey and Haus 2018). Reactive carbonyl species (RCS) [e.g., methylglyoxal (MGO)] and reactive aldehydes [e.g., 4-hydroxynonenal (4-HNE)]; positively associate with disease severity and are elevated in the post-prandial and hyperinsulinaemic state (Mey et al. 2018; Neri et al. 2010). MGO and 4-HNE can directly interfere with insulin signalling in skeletal muscle (Pillon et al. 2012; Riboulet-Chavey et al. 2006) and form adducts with proteins, which modifies their activity and leads to the downstream formation of harmful advanced lipid peroxidation (ALEs) and advanced glycation (AGEs) end-products—further exacerbating T2D and diabetic complications (for a review, see Brings et al. 2017). There is, therefore, a need to develop novel interventions that reduce oxidative and carbonyl stress to help delay or prevent disease development and progression.

Carnosine (*β*-alanyl-L-histidine) is a multifunctional dipeptide with an emerging role in metabolic health and disease (Artioli et al. 2019). It exists in high concentrations in human skeletal muscle (approx. 22 mmol kg^−1^ dw^−1^; 5 mmol kg^−1^ ww^−1^), which can be increased by up to twofold with prolonged supplementation of its rate-limiting precursor, *β*-alanine (Matthews et al. 2019; Saunders et al. 2017). Dietary sources include prawns, tuna, mackerel, poultry, and red meats; providing ~ 300–550 mg^.^day^−1^ *β*-alanine (Baguet et al. 2011; Kratz et al. 2017). Importantly, our recent meta-analysis suggested that supplementation with carnosine or *β*-alanine reduces fasting glucose and glycated haemoglobin (HbA_1c_) in humans and rodents (Matthews et al. 2021). While this is promising, there are issues with risk of bias and study quality, and the site and mechanism of action remain unclear. The beneficial effects could be due to carnosine forming stable adducts with reactive species, thereby reducing their reactivity and allowing them to be safely metabolised or excreted from the body (Baba et al. 2013; Regazzoni et al. 2016; Szwergold 2005). Consistent with this, evidence from human and rodent studies shows that carnosine supplementation protects against oxidative stress, lipid peroxidation, and AGE and ALE formation (Albrecht et al. 2017; Aldini et al. 2011; Elbarbary et al. 2018; Houjeghani et al. 2018).

Mechanistic research from our group showed that C2C12 skeletal muscle cells cultured under glucolipotoxic (GLT) conditions (a model of metabolic stress) had reduced glucose uptake and higher reactive oxygen species (ROS) (Cripps et al. 2017), as well as a suppression of GLUT4 translocation and cellular respiration (ATP-linked and maximal O_2_ consumption) (Lavilla et al. 2021). In both studies, treatment with 10 mM carnosine led to a partial recovery of glucose uptake and GLUT4 translocation, a near full recovery of cellular respiration, and normalisation of ROS (Cripps et al. 2017; Lavilla et al. 2021). Consistent with its role as a scavenger of reactive species, carnosine prevented 90% and 80% of 4-HNE protein adduction in C2C12 and human skeletal muscle cells (Lavilla et al. 2021). It is also possible that carnosine acts indirectly by activating endogenous anti-carbonylation, e.g., glyoxalase 1 (GLO1) defence systems (Aldini et al. 2021), or by increasing the expression of metabolic proteins involved in regulating mitochondrial health e.g., NAD-dependent deacetylase sirtuins 1/3 (Sirt1, Sirt3) or peroxisome proliferator-activated receptor gamma coactivator a-alpha (PCG-1α), which have been linked to improvements in insulin signalling and oxidative stress (Jing et al. 2011; Pagel-Langenickel et al. 2008). These actions, coupled with the ability to increase tissue carnosine stores through diet, makes it a promising therapeutic for T2D and prediabetes.

Herein we extend our previous work by reporting novel investigations on the effect of carnosine and *β*-alanine on cellular respiration, glucose uptake, and carbonyl and aldehyde-modified proteins in primary T2D and healthy human skeletal muscle cells.

## Methods

Cell culture work was performed in a Class II laminar flow hood under aseptic conditions. For subculture and experiments, cells were kept at 37 °C and 5% CO_2_ in a humidified incubator, and all reagents and plasticware were purchased from Thermo Fisher Scientific (Loughborough, UK), unless otherwise stated. Growth, differentiation, and treatment media were changed every other day or as part of general sub-culture procedures.

### Human skeletal muscle cell culture

Human skeletal myoblasts isolated from lean healthy control (LHC; male, 20y, BMI 21 kg^.^m^2^, lot no. 639629) and obese type-2 diabetic (T2D; male, 68y, BMI 33 kg^.^m^2^, lot no. 211384) donors were purchased from Lonza Bioscience (Basel, Switzerland). Cells were pre-screened to display ≥ 60% desmin-positive cells at first passage and tested negative for mycoplasma, bacteria, yeast, virus, and fungi. Myoblasts were cultured in skeletal muscle growth medium (PromoCell, Germany): basal medium, 5.5 mM glucose, supplemented with 10% fetal bovine serum (FBS), 50 µg/mL bovine fetuin, 10 µg/mL human insulin, 10 ng/mL human epidermal growth factor, 1 ng/mL human basic fibroblast growth factor, and 0.4 µg/mL dexamethasone. Myoblasts were differentiated to myotubes by changing growth medium to DMEM:F-12 supplemented with 2% horse serum and 0.5% penicillin–streptomycin for 4 to 5 days. Experiments were performed with cells at passages 3–7 due to reduced myogenic potential at higher passages.

Myotubes were treated for 4 days with vehicle (control), 10 mM carnosine, or 10 mM *β*-alanine (Sigma-Aldrich, UK) dissolved in low-glucose DMEM, DMEM:F-12, or GLT DMEM:F-12. GLT media was used for some experiments as an extracellular model of poorly controlled type-2 diabetes (DMEM:F-12 containing 17 mM glucose with added 200 µM palmitic acid and 200 µM oleic acid). DMEM:F12 was supplemented with 2% fatty acid free bovine serum albumin (BSA); stock solutions of 100 mM palmitic acid, dissolved in 100% ethanol, and 100 mM sodium oleate, dissolved in 50% ethanol, were heated to 60 °C and added directly to the BSA (Sigma-Aldrich, UK). The media was then incubated at 37 °C for 1 h to allow fatty acid conjugation, before being sterile filtered through 0.2 µm membrane filters (Lavilla et al. 2021; Marshall et al. 2007).

### Cell viability

Cell viability was measured using alamarBlue™. Cells were seeded at 2 × 10^4^ cells/cm^2^ in 24-well plates, differentiated, treated for 4-days, then washed with Dulbecco’s phosphate buffered saline (DPBS) and incubated with differentiation medium ± 10% (v/v) alamarBlue™ for 1-h at 37 °C and 5% CO_2_. Samples were transferred to black 96-well plates and fluorescence recorded (excitation 570 nm, emission 585 nm) on a spectrophotometer (CLARIOstar Plus, BMG Labtech, Germany). Results were corrected for background fluorescence by subtracting negative control wells from experimental wells and are expressed as fold change relative to control wells.

### Measurement of O_2_ consumption

Oxygen consumption rate (OCR) was measured using a Seahorse XFe24 Analyser and XF Cell Mito Stress Test reagents (Seahorse Bioscience Inc., USA). Myoblasts were seeded at 3 × 10^4^ cells per well in XF24 polystyrene V7 microplates, differentiated, and treated for 4-days. The day before the assay, a Seahorse XF sensor cartridge was hydrated with XF pH 7.4 calibrant and kept in a 37 °C non-CO_2_ incubator overnight. For the assay, cells were washed with DPBS and treated with bicarbonate-free Seahorse XF DMEM supplemented with 1 mM pyruvate, 2 mM glutamine, and 10 mM glucose, and kept in a 37 °C non-CO_2_ incubator for 45 min. Carnosine, *β*-alanine, or vehicle (control) were added to the assay media and pH adjusted to 7.4 ± 0.05. To reduce common variability issues, we allocated 6–7 wells per condition per assay. Metabolic inhibitors were dissolved in assay media and loaded into the sensor cartridge using concentrations that were optimised for each cell type in prior assays.

Baseline OCR was measured 4 times for 3 min. Following which, 0.8 µM oligomycin was added to each well to quantify state four (non-phosphorylating) respiration and OCR was measured 3 times for 3 min. Then, 5 µM carbonyl cyanide 4-(trifluoromethoxy)phenylhydrazone (FCCP) was added to each well to quantify maximal respiration and OCR was measured 4 times for 3 min each. For the final step, 0.5 µM rotenone/antimycin A was added to each well to quantify non-mitochondrial respiration and OCR was measured 3 times for 3 min each. All measurements were separated by a 2-min wait and 2-min mix. Once completed, assay media was aspirated and protein extracted from each well, which was used to normalise OCR values (pmol^.^min^−1.^µg total protein). Included wells were corrected for background OCR by subtracting negative control wells and averaged to provide a single OCR value at each measurement point for each condition. Total area-under-the-curve for O_2_ consumption was measured as previously described (Narang et al. 2020).

### Measurement of glucose uptake

Glucose uptake was measured in myotubes using a bioluminescent assay based on the detection of 2-deoxyglucose-6-phosphate (2DG6P) (Promega, UK). Cells were seeded at 2 × 10^4^ cells/cm^2^ in 24-well plates, differentiated, treated for 4-days, and serum-starved overnight in low-glucose 5.5 mM DMEM ± treatment. For the assay, cells were washed with DPBS and incubated with glucose-free DMEM ± 100 nM human insulin (Sigma-Aldrich, UK) for 1-h at 37 °C and 5% CO_2_. The media was then replaced with DPBS containing 200 µM 2DG for 30 min at 37 °C and 5% CO_2_. For negative control wells 50 µM of cytochalasin B (Sigma-Aldrich, UK), an inhibitor of GLUT-dependent glucose uptake, was added 5 min before the addition of 2DG. Following incubation, 0.4 M HCl + 2% dodecyl trimethyl ammonium bromide (stop buffer) was added to lyse cells, terminate uptake, and eradicate intracellular NADPH, then a high-pH buffer solution (neutralisation buffer) added. For the final step, the 2DG6P detection reagent was added to each well and incubated at room temperature for 1-h. Data were acquired for luminescence using a 0.3–1 s integration on a spectrophotometer (CLARIOstar Plus, BMG Labtech, Germany). Negative control wells were subtracted from experimental wells and relative light units converted to glucose uptake (nmol min^−1^) using a 2DG standard curve after correcting for assay duration and dilution factor.

### Protein extraction and measurement

Myotubes were washed with DPBS and extracted using a cell scraper and centrifuged for 5 min at 300*g*. Cells were then incubated with ice-cold radioimmunoprecipitation buffer (Pierce^®^, Thermo Fisher, UK) with a protease inhibitor cocktail (Halt™, Thermo Fisher, UK); agitated for 20 min at 4 °C and centrifuged for 15 min at 4 °C and 13,000*g*. Protein content was determined via bicinchoninic acid assay with absorbance recorded at 562 nm using a spectrophotometer (CLARIOstar Omega, BMG Labtech, Germany). Negative control wells were subtracted from experimental wells; and data normalised to standard curve values.

Protein expression was measured via Western blotting SDS-PAGE (sodium dodecyl sulfate-polyacrylamide gel electrophoresis) according to published recommendations (Bass et al. 2017; Mahmood and Yang 2012). Equipment and consumables were from Bio-Rad (Bio-Rad Laboratories Inc., UK), unless otherwise stated. Approx. 20–25 µg total denatured proteins were separated in precast gels with running buffer (25 mM Tris, 190 mM glycine, 0.1% SDS) and transferred to methanol-activated PVDF membranes using a Trans-Blot Turbo system with transfer buffer (25 mM Tris, 190 mM glycine, 20% ethanol; 0.1% SDS was added for proteins larger than 80 kDa). Membranes were blocked (3% non-fat milk in TBST) at room temperature for 1-h, before incubation with primary antibodies (diluted in 3% BSA in TBST) at 4 °C overnight. The following morning, membranes were incubated with HRP (horseradish-peroxidase)-conjugated secondary antibody (diluted in 5% non-fat milk in TBST) at room temperature for 1 h. To measure multiple proteins of a similar molecular weight, membranes were stripped and re-probed using a mild stripping buffer (200 mM glycine, 0.1% SDS, 1% Tween 20, pH 2.2). Membranes were washed in Tris-buffered saline with Tween 20 (TBST) between each step. Primary antibodies: MGO (ab243074), LDHA (ab84716), Sirt1 (ab110304), Sirt3 (ab45067) (Abcam, Cambridge, MA); GLO1 (sc-133214), PGC-1α (sc-517380) (Santa Cruz Biotechnologies, Santa Cruz, CA); 4-HNE (ab46545). Secondary antibodies (Bio-Rad Laboratories Inc., UK) were selected in a species-specific manner, according to primary antibody instructions.

For imaging, each membrane was incubated with Clarity Max enhanced chemiluminescence substrate for 5 min and imaged using the GeneGnome XRQ and GeneSys image acquisition software version 1.7.2. (SynGene, Cambridge, UK). Semi-quantitative densitometry analysis of blots was performed using Image J (version 1.8.0) and data normalised to *β*-actin expression. MGO and 4-HNE-modified proteins were measured using the range of detected bands between 10 and 260 and 20 and 100 kDa, an approach used previously in human skeletal muscle tissue (Mey et al. 2018). Data are presented as the fold change relative to control.

### Statistical analysis

Results are presented as mean ± standard error of the mean (SEM) (*n* = 3 or more independent experiments). Data were analysed for each experiment using a one-way ANOVA with post hoc comparisons using the Tukey (glucose uptake, protein adduction, ATP-linked, and maximal respiration) or the Bonferroni method (cell viability and OCR tAUC), with *p* values adjusted for multiple comparisons. Differences between absolute basal (LHC vs T2D) and absolute insulin-stimulated (LHS vs T2D) glucose uptake were analysed using two-tailed unpaired t-tests. Analyses were performed using Prism v9.1.0 (GraphPad Software, USA); with *p*-values < 0.05 considered statistically significant.

## Results

### Cell viability

There was no significant effect of treatment on cell viability (Supplementary Fig. 1). Because of this, the 10 mM treatment concentration was used for subsequent experiments, as this is near to the upper limit of intramuscular skeletal muscle carnosine stores after supplementation with its precursor, *β*-alanine, in vivo (Saunders et al. 2017).

### Cellular respiration

**Fig. 1:**
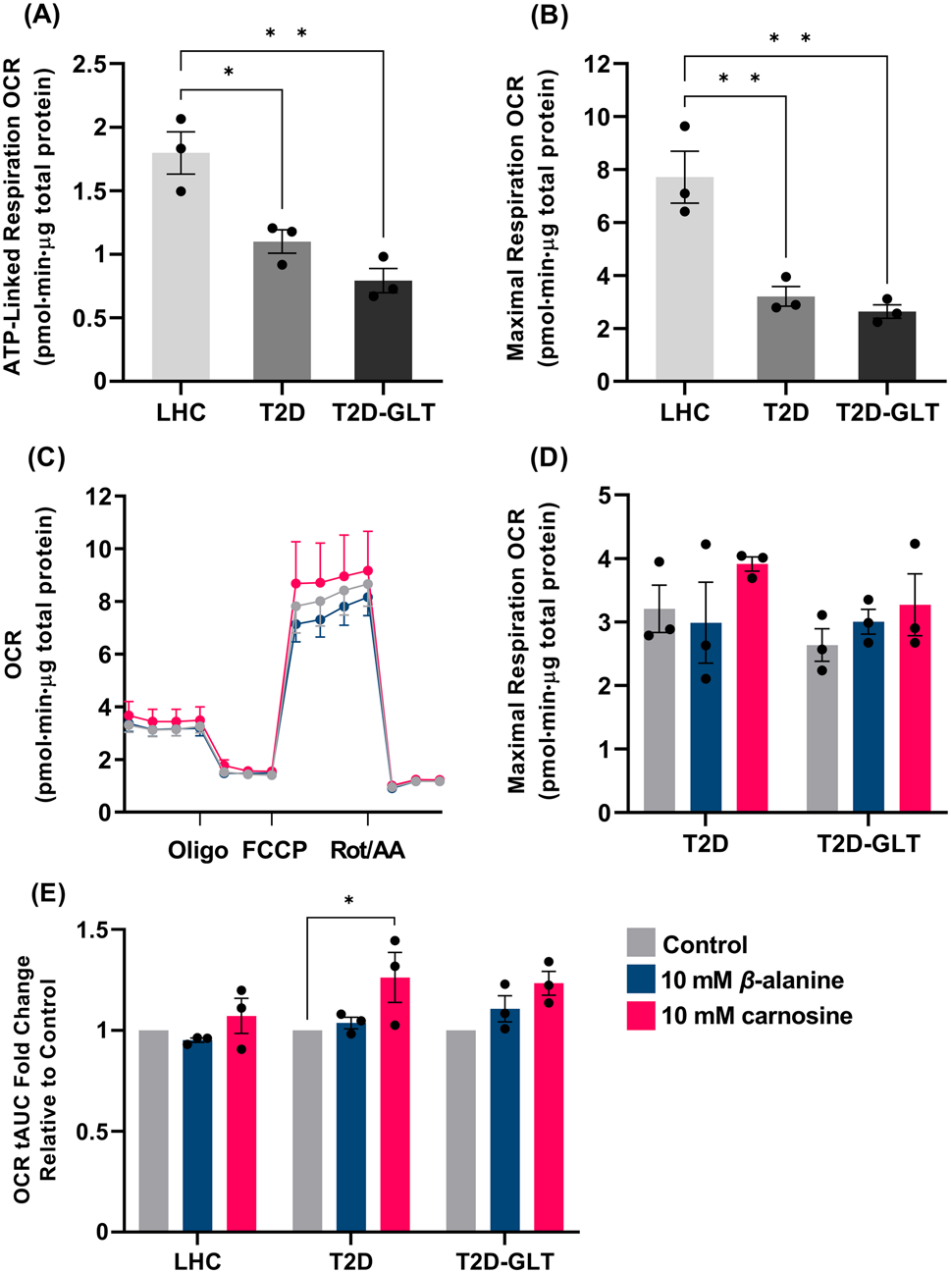
Skeletal muscle O_2_ consumption measured in human skeletal myotubes using the Seahorse XFe24 Analyser. **A**, **B** ATP-linked and maximal respiration for all cell conditions. **C** Seahorse Mito Stress Test trace for LHC cells. **D** Maximal respiration for T2D and T2D-GLT cells. **E** OCR tAUC for all cell conditions, showing the fold-change relative to each control condition (*n* = 3 independent experiments per condition, *n* = 6–7 replicates per experiment). *p < 0.05, **p < 0.01. *LHC* lean healthy control, *OCR* O_2_ consumption rate, *T2D-GLT* type-2 diabetic glucolipotoxic conditions, *tAUC* total area-under-the-curve

T2D and T2D-GLT cells had significantly reduced ATP-linked and maximal respiration compared with LHC cells (T2D *p* = 0.016 and *p* = 0.005; T2D + GLT *p* = 0.0028 and *p* = 0.003; Fig. 1A, B). There were no statistically significant differences between T2D and T2D + GLT. ATP-linked and maximal respiration were not statistically affected by treatment, although there was a small, non-significant increase in all cells treated with carnosine (Fig. 1C, D). Carnosine increased total O_2_ consumption area-under-the-curve relative to control in T2D cells (*p* = 0.035, T2D-GLT cells; *p* = 0.073, Fig. 1E).

### Glucose uptake

**Fig. 2:**
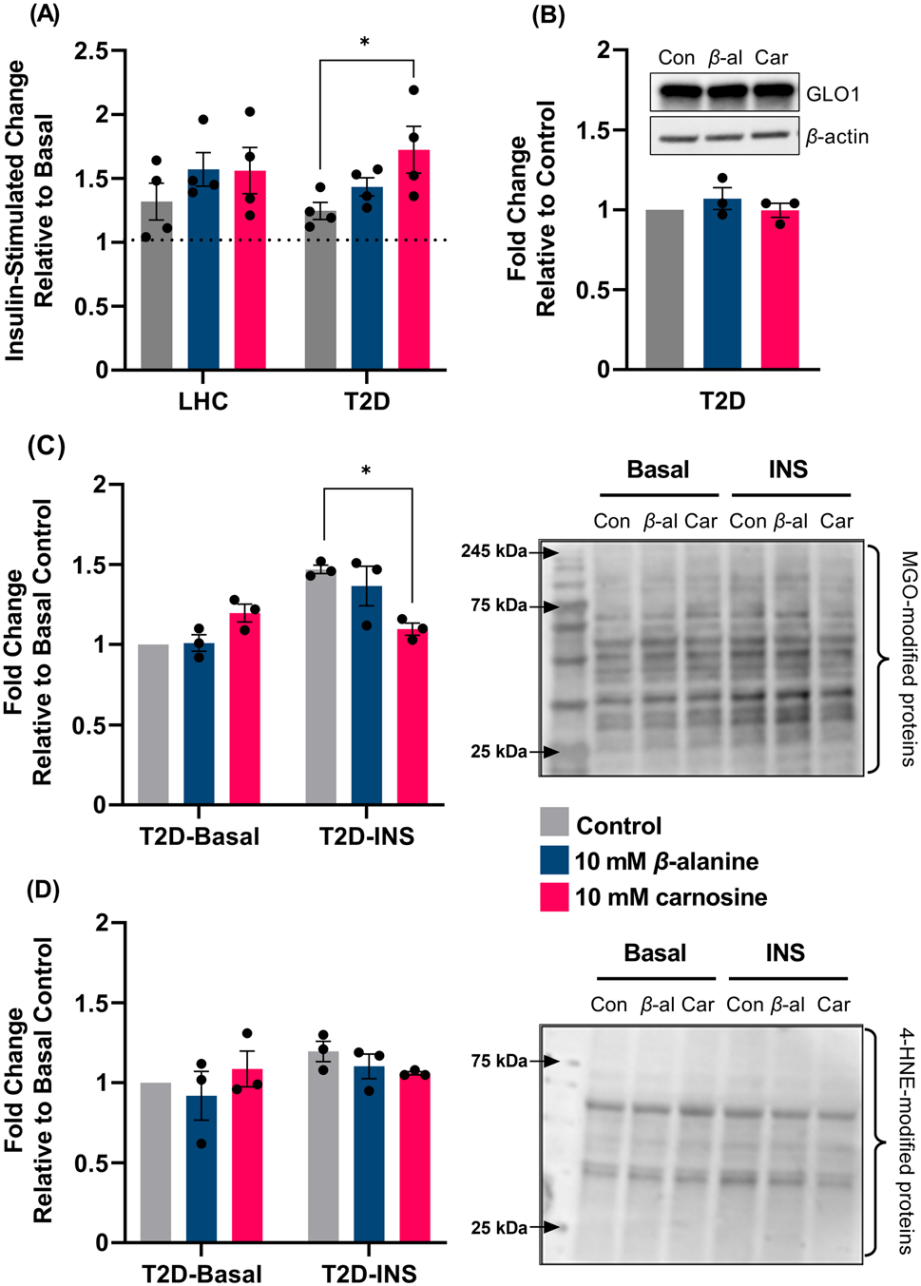
Insulin-stimulated glucose uptake and protein adducts in human skeletal myotubes. **A** Glucose uptake in LHC and T2D; **B** GLO1 expression in T2D myotubes; **C** MGO-modified proteins in T2D cells under basal and insulin-stimulated (INS) conditions; **D** 4-HNE-modified proteins in T2D cells under basal and INS conditions (*n* = 3 independent experiments per condition, *n* = 3–4 replicates per experiment). *p < 0.05. *β-al* *β*-alanine, *Car* carnosine, *LHC* lean healthy control, *OCR* O_2_ consumption rate, *T2D* type-2 diabetic

Absolute basal and insulin-stimulated glucose uptakes were significantly lower in T2D cells compared with LHC cells (basal: 17.66 ± 1.56 vs. 51.36 ± 2.57 nmol^.^min^−1^; *p* < 0.0001, insulin-stimulated: 22.11 ± 2.62 vs. 66.89 ± 5.31 nmol^.^min^−1^; *p* = 0.0003). Treatment with carnosine significantly increased the ratio of basal to insulin-stimulated glucose uptake in T2D cells (*p* = 0.047, Fig. 2A); treatment did not affect LHC cells.

### Protein expression and modification

Insulin-stimulation increased MGO-modified proteins in T2D cells by 47%; treatment with carnosine attenuated this increase to 9.7% (*p* = 0.011; Fig. 2C). There was no effect of insulin-stimulation or treatment on 4-HNE modified proteins in T2D cells (Fig. 2D); and no effect of treatment on expression of GLO1 (Fig. 2B) or other metabolic proteins (LDHA, PCG-1α, Sirt1, or Sirt3; Supplementary Fig. 2).

## Discussion

Our main finding is that treatment of primary T2D skeletal muscle cells with carnosine increases insulin-stimulated glucose uptake. This occurred in parallel with a reduction in insulin-stimulated MGO-modified proteins, which is a possible mechanism of action. To our knowledge, this is the first study to show these effects in T2D skeletal muscle cells. Our data also show that T2D skeletal muscle cells have significantly lower ATP-linked respiration, maximal respiration, and basal and insulin-stimulated glucose uptake when compared with LHC cells. This preservation of the T2D phenotype validates our cell model, which is consistent with previous work in human myotubes (Gaster 2019).

The increase in insulin-stimulated glucose uptake occurred independently of changes in cell viability and this finding supports our previous work in mouse C2C12 skeletal muscle cells under metabolic stress (Cripps et al. 2017). Herein, we showed a 47% increase in MGO-modified proteins following insulin-stimulation, which is comparable to the increase seen in T2D muscle following a hyperinsulinaemic–euglycaemic clamp (Mey et al. 2018). Treatment with carnosine attenuated the increase in MGO-modified proteins to 9.7%, which could explain the beneficial effects on glucose uptake. In T2D skeletal muscle, insulin-stimulated glycolytic flux leads to MGO formation via the spontaneous oxidation of glycolytic intermediates glyceraldehyde-3-phosphate (G3P) and dihydroxyacetone phosphate (DHAP) (Mey et al. 2018; Phillips and Thornalley 1993). Excessive MGO production affects glucose transport and insulin signalling in a dose-dependent manner by binding directly to insulin receptor substrate (IRS) proteins and altering their structure and function (Riboulet-Chavey et al. 2006). Under normal conditions, MGO and downstream AGE formation is balanced by detoxification through the GLO1 enzyme system (Rabbani and Thornalley 2012). As GLO1 expression is reduced in T2D skeletal muscle (Mey et al. 2018), we explored whether the reduction in MGO-modified proteins might have occurred as a result of a change in GLO1 expression. GLO1 was unaffected by carnosine or *β*-alanine, which points towards a direct role of carnosine binding to MGO, thereby reducing MGO-mediated protein modification, as shown previously (Hipkiss and Chana 1998). Some studies, however, have suggested that carnosine has only low scavenging activity towards MGO (Colzani et al. 2016; Vistoli et al. 2017). This suggests that carnosine could potentially have reduced MGO-protein modification indirectly by activating endogenous antioxidant and anti-carbonylation defence systems via the nuclear factor erythroid 2-related factor 2 (Nrf2) signalling cascade (for a detailed review, see Aldini et al. 2021) and these mechanisms should be explored in future studies.

Carnosine is an efficient scavenger of the reactive aldehyde, 4-HNE (Colzani et al. 2016); and our previous work showed carnosine prevents 4-HNE protein adduction events in human plasma and human skeletal muscle cells (Lavilla et al. 2021). Despite this, we did not see a reduction in basal or insulin-stimulated 4-HNE-modified proteins in the present study. There are several possible explanations for this. Firstly, while plasma 4-HNE increases ~ 33% in the postprandial state in people with T2D (Neri et al. 2010), this might not translate into an increase in intramuscular 4-HNE within the 1-h insulin-stimulation period used in our study. Further, our previous work involved cells being cultured in GLT conditions for 5-days, which suggests that a chronic hyperglycaemic environment is important for generating intracellular 4-HNE. In further support of this assertion, Mey et al. (2018) showed that global carbonyl stress was only ~ 11% higher in T2D skeletal muscle under basal conditions and was not affected by a hyperinsulinaemic–euglycaemic clamp.

Our data show an increase in total-area-under-the-curve O_2_ consumption in T2D cells treated with carnosine; T2D-GLT cells followed a similar pattern but did not reach statistical significance. The increase in O_2_ consumption was from mitochondrial and non-mitochondrial respiration and reflects a general increase in cell respiratory capacity (Mitov et al. 2017). These results should be considered alongside the absence of significant changes in ATP-linked and maximal respiration, which are the markers that best-reflect changes in mitochondrial content and oxidative capacity (Divakaruni et al. 2014). This is supported by the lack of change in protein expression linked to mitochondrial biogenesis. These findings are in contrast to previous work in C2C12 myotubes, where treatment with 800 µM *β*-alanine induced several markers of mitochondrial biogenesis and increased O_2_ consumption (Schnuck et al. 2016); and work in rat cardiomyocytes, where treatment with 5 mM *β*-alanine caused mitochondrial fragmentation and oxidative stress, leading to a reduction in cellular O_2_ consumption (Shetewy et al. 2016). Our results suggest that treatment with 10 mM *β*-alanine does not cause these beneficial or deleterious changes in human skeletal muscle. The addition of GLT media to T2D cells had only a negligible effect on cellular respiration. This is in contrast to our previous work that showed a substantial reduction in ATP-linked and maximal respiration in LHC cells under GLT conditions (Lavilla et al. 2021). We take this to show that the extracellular environment has less influence on T2D cellular function due to the inherent defects that persist in vitro, whereas defects can be induced in the LHC cells, and a recovery of function made, owing to their original healthy state.

Our study raises several questions that warrant further investigation. We did not see an effect for *β*-alanine alone on any outcome, which could be due to insufficient time or substrate for carnosine synthesis (DMEM:F-12 culture media contains ~ 203 µM histidine). Because of this, our results suggest that the beneficial effects shown are due to the intact dipeptide carnosine, and not its rate-limiting precursor, although a combined *β*-alanine and histidine treatment group would be needed to confirm this. We did not directly measure carnosine adducts, or carnosine-binding to MGO, meaning our results are associative, and several other pro-inflammatory reactive species (e.g., malondialdehyde, 3-nitrotyrosine, acrolein, and 4-hydroxy-2-hexanal) could be implicated. Future research should build on this and characterise the effect of carnosine on the insulin signalling cascade by measuring the phosphorylation status of relevant proteins (e.g., Akt and TBC1D4), stress-activated kinase signalling (e.g., AMPK, p38, and CAMKII), and GLUT4 translocation.

Data presented herein indicate that carnosine can increase insulin-stimulated glucose uptake and reduce insulin-stimulated MGO-modified proteins in T2D skeletal muscle cells. This expands upon our previous work showing beneficial effects on protein adduction in human cells and glucose uptake in C2C12 cells. These results have implications for the role of carnosine in T2D and prediabetes and suggest that the beneficial effects on glycaemic control in human clinical trials may be explained by its RCS-scavenging actions in human skeletal muscle.

## Supplementary Information

Below is the link to the electronic supplementary material.

Supplementary file1 (DOCX 231 KB)

## Data Availability

The datasets generated during and/or analysed during the current study are available from the corresponding author on reasonable request.
