# Supplementary material for: Carnosine increases insulin-stimulated glucose uptake and reduces methylglyoxal-modified proteins in type-2 diabetic human skeletal muscle cells
Source: Amino Acids. 2023 Jan 13;55(3):413–20. doi: 10.1007/s00726-022-03230-9 (PMC10038967; doi:10.1007/s00726-022-03230-9)
Supplement: Supplementary file 1 — Supplementary file1 (DOCX 231 KB) [file 726_2022_3230_MOESM1_ESM.docx]

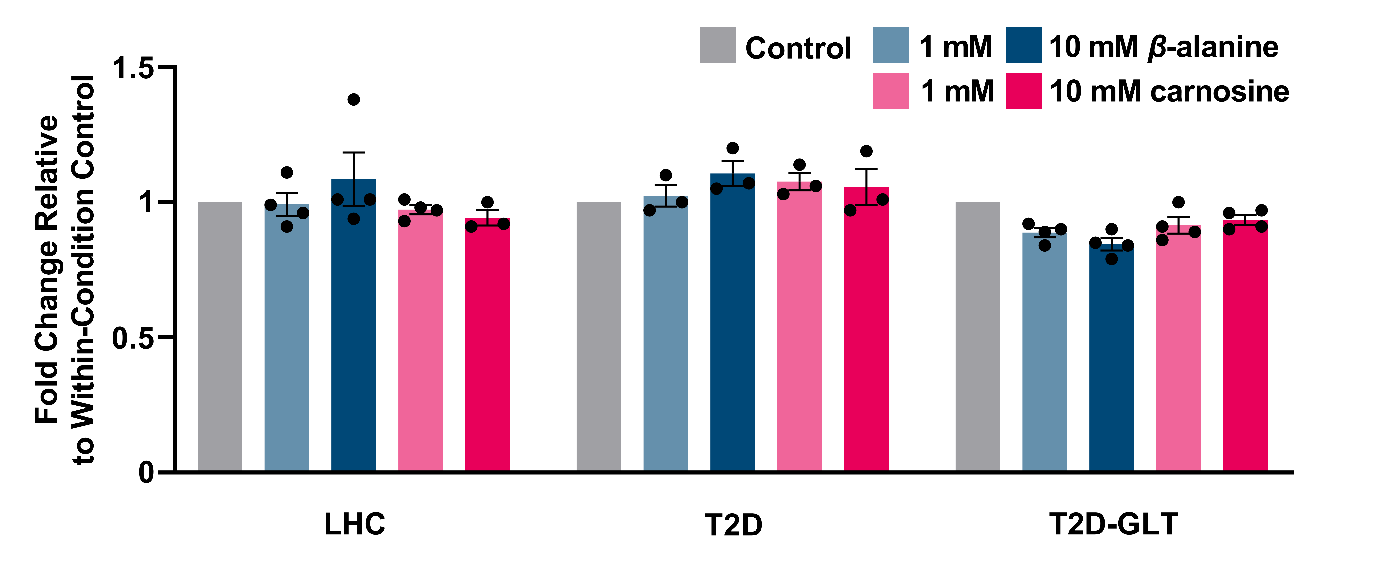


**Supplementary Figure 1.** Cell viability measured in human skeletal myotubes using the alamarBlue^TM^ reagent in after 4-days of treatment (*n* = 3-4 independent experiments per condition, *n* = 3-4 replicates per experiment).


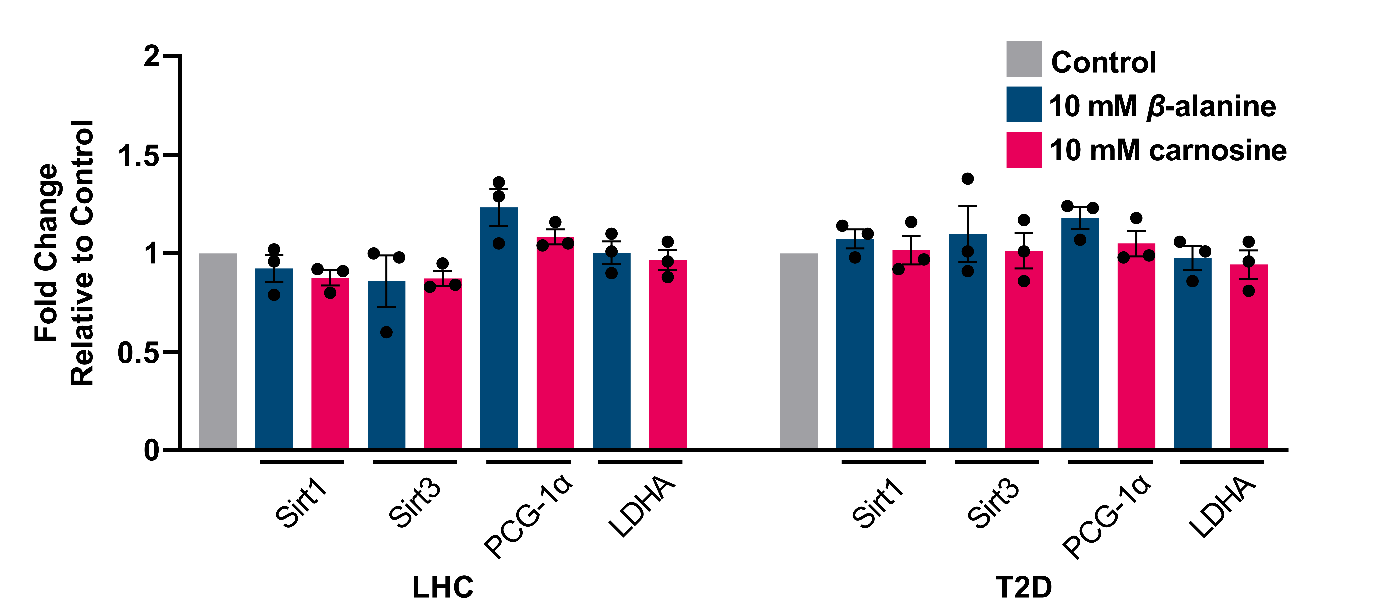


**Supplementary Figure 2.** Protein expression in human skeletal myotubes measured via Western blotting SDS-PAGE (*n* = 3 independent experiments per condition, *n* = 3-4 replicates per experiment). **p*<0.05. LDHA; lactate dehydrogenase A, LHC; lean healthy control, PCG-1α; peroxisome proliferator-activated receptor gamma coactivator a-alpha, Sirt1/3; NAD-dependent deacetylase sirtuins 1/3, T2D; type-2 diabetic.
